# Development of a MALDI MS‐based platform for early detection of acute kidney injury

**DOI:** 10.1002/prca.201500117

**Published:** 2016-05-17

**Authors:** Emma Carrick, Jill Vanmassenhove, Griet Glorieux, Jochen Metzger, Mohammed Dakna, Martin Pejchinovski, Vera Jankowski, Bahareh Mansoorian, Holger Husi, William Mullen, Harald Mischak, Raymond Vanholder, Wim Van Biesen

**Affiliations:** ^1^ Institute of Cardiovascular and Medical SciencesGlasgowUK; ^2^ Renal DivisionGhent University HospitalBelgium; ^3^ Mosaiques Diagnostics GmbHHannoverGermany; ^4^ Charite‐Universitätsmedizin BerlinBerlinGermany; ^5^ RWTH AachenInstitute of Molecular Cardiovascular ResearchAachenGermany

**Keywords:** Acute kidney injury, MALDI‐MS, Peptide marker model

## Abstract

**Purpose:**

Septic acute kidney injury (AKI) is associated with poor outcome. This can partly be attributed to delayed diagnosis and incomplete understanding of the underlying pathophysiology. Our aim was to develop an early predictive test for AKI based on the analysis of urinary peptide biomarkers by MALDI‐MS.

**Experimental design:**

Urine samples from 95 patients with sepsis were analyzed by MALDI‐MS. Marker search and multimarker model establishment were performed using the peptide profiles from 17 patients with existing or within the next 5 days developing AKI and 17 with no change in renal function. Replicates of urine sample pools from the AKI and non‐AKI patient groups and normal controls were also included to select the analytically most robust AKI markers.

**Results:**

Thirty‐nine urinary peptides were selected by cross‐validated variable selection to generate a support vector machine multidimensional AKI classifier. Prognostic performance of the AKI classifier on an independent validation set including the remaining 61 patients of the study population (17 controls and 44 cases) was good with an area under the receiver operating characteristics curve of 0.82 and a sensitivity and specificity of 86% and 76%, respectively.

**Conclusion and clinical relevance:**

A urinary peptide marker model detects onset of AKI with acceptable accuracy in septic patients. Such a platform can eventually be transferred to the clinic as fast MALDI‐MS test format.

**Abbreviations**
AKI: acute kidney injury
AUC: area under the ROC curve
B2M: beta‐2 microglobulin
CI: confidence interval
CKD: chronic kidney disease
ICU: intensive care unit
NGAL: neutrophil gelatinase‐associated lipocalin
RIFLE: risk/injury/failure/loss of kidney function/end stage renal disease
ROC: receiver operating characteristics
SVM: support vector machine

## 1 Introduction

The reported incidence of acute kidney injury (AKI) associated with sepsis is steadily increasing, estimated at 2.8% per annum 1. This is due to an increase of comorbidity conditions and the severity of illness 1. Whereas in hospital mortality rate for AKI decreased from 41.3% in 1988 to 28.1% in 2002 2, there is an increasing concern that AKI is associated with later development of chronic kidney disease (CKD) 3, 4, 5, 6, 7.

**Clinical Relevance**

There is a clinical need for new biomarkers enabling a more accurate and timely detection of septic acute kidney injury (AKI) which in turn might lead to an improvement in both short and long‐term outcome. The reported incidence of sepsis and hence septic AKI is steadily increasing. However, relatively little progress has been made in its management, and outcome remains grim. Relying on existing single component biomarkers for early AKI diagnosis, has proven to be disappointing because these single biomarkers are unable to capture the complex pathophysiological process of septic AKI. A panel of biomarkers might be more appropriate for this purpose and allow for earlier diagnosis of septic AKI compared to single biomarkers and standard parameters such as serum creatinine and urinary output. We have previously identified a panel of biomarkers by using CE‐MS. By using this panel, we could detect AKI 5 days earlier than existing biomarkers. However, CE‐MS is a highly specialized research tool that is not suited for daily clinical practice. Our aim was to develop an early predictive test for AKI based on the analysis of urinary peptide biomarkers by MALDI‐MS. Such a platform can be transferred to the clinic as point‐of‐care test to provide timely detection of septic AKI to improve clinical outcome.

The severity of AKI also has a direct effect on the outcomes and even small increases in serum creatinine levels are inversely related to long‐term survival of patients, length of hospital stay, and costs 8. Overall, little progress has been made in AKI management and its associated complications so that the overall outcome remains grim 1, 9, 10.

There are several explanations for the lack of progress in the field of septic AKI. Serum creatinine only starts to rise hours after the initial insult to the kidney 11. Moreover, it is a functional parameter that can also increase physiologically, e.g. in case of dehydration. During sepsis, creatinine production is reduced, and the distribution volume increased due to capillary leakage and fluid overload 12, which can further add to the delay in diagnosis 13. These observations have encouraged the quest for new biomarkers that might allow diagnosis at an earlier stage. However, results have been inconsistent 14, 15, and it is still unclear whether these biomarkers do have any relevance in clinical management of patients.

The pathophysiology of septic AKI is largely unknown 16, 17, 18. In addition, in most cases, there is no clear timing of renal insult. The appearance of biomarkers is time related and thus the window for biomarker detection can easily be missed. Therefore, there might be no single biomarker able to capture the complex pathophysiological processes of septic AKI. A clinical review of 12 existing biomarkers for AKI including cystatin C, neutrophil gelatinase‐associated lipocalin (NGAL), and kidney injury molecule 1 found their discriminatory power to be poor, with AUC's ranging from 0.5 to 0.8 2. A panel of biomarkers might offer advantages for AKI detection 19. Such a panel might offer a better pathophysiological understanding and provide insights in the pathophysiological link between AKI and later CKD, which affects a substantial proportion of AKI patients 4, 20.

We have previously demonstrated that by using CE‐MS we could identify a urinary peptide marker profile that performed better than traditional and single biomarkers for early AKI diagnosis in intensive care patients 19. The drawback of CE‐MS is that the time requirement (>24 h), the high skill operating level and high cost make it impractical for close monitoring of acute events and point of care testing in daily clinical practice. Analysis of peptides can also be carried out using MALDI‐MS that is a higher throughput, less expensive and less skilled approach 21. Hence, the MALDI‐MS platform is currently more suitable than CE‐MS for the implementation of fast screening assays in daily clinical practice providing a test result within a few hours.

In this study, we aimed to use the MALDI‐MS platform in a cohort of sepsis patients to identify a marker panel for timely AKI diagnosis. This might lead to a better outcome in sepsis patients, by limiting fluid overload 22, 23 and preventing further renal damage through avoidance of nephrotoxic drugs 24.

## 2 Materials and methods

### 2.1 Patients and samples

The urinary low molecular weight proteome of 95 out of 195 septic patients enrolled in the clinical trial NCT01981993 and admitted to the Ghent University Hospital between 12/01/2010 and 27/03/2011 was analyzed by MALDI‐MS. For patient selection in this nested case–control study, patients of the original study were grouped into AKI cases and controls and within these groups randomly selected for MALDI analysis using the random sample generator of MedCalc 11.4.1.0 (Mariakerke, Belgium). We defined sepsis, severe sepsis and septic shock according to the American College of Chest Physicians/Society of Critical Care Medicine Consensus Conference 25. AKI was defined according to the risk, injury, failure, loss of kidney function, and end‐stage renal disease (RIFLE) guidelines 26 based on both serum creatinine and urinary output criteria within the first 5 days after admission. Written informed consent was obtained from all patients and ethical approval was obtained from the Ghent University Hospital ethics committee. The study conformed to the Helsinki Declaration standards.

Urine samples were collected from the urinary catheter within 12 hours after intensive care unit (ICU) admission and after transfer to a 10 mL‐Monovette immediately frozen and stored at –80°C until analysis. From the 95‐patient set, 17 patients with and 17 patients without onset of AKI were randomly selected as a training group for statistical AKI biomarker search and multimarker model establishment, whereas all remaining patients were used as test groups for validation of the peptide marker model. Clinical and demographic characteristics of the AKI and non‐AKI patients included in the training and test groups are given in Table 1.

**Table 1:** Clinical and demographic data of the sepsis patients with (case group) or without (control group) AKI progression included in the training and test sets of the case–control study for MALDI AKI model establishment

Parameter	Training sets			Test sets		
	AKI	Non‐AKI	*p* b)	AKI	Non‐AKI	*p* b)
Patients/samples (n)	17/17	17/17		44/44	17/17	
Age (years)a)	62 (39–81)	58 (18–83)	0.65	62 (24–89)	57 (17–77)	0.33
Gender male (%)	52.9	64.7	0.73	40.9	52.9	0.57
APACHE II score during the first 24h after ICU admissona)	22 (15–32)	20 (12–36)	0.24	25 (9–41)	19 (1–28)	0.03
Serum creatinine at ICU admission (mg/dL)a)	0.83 (0.42–1.32)	0.85 (0.34–1.79)	0.58	0.85 (0.27–2.20)	0.90 (0.55–1.31)	0.32
Historical baseline CKD‐EPI (ml/min/1.73m^2^)a)	87.3 (55.8–124.2)	95.7 (38.4–149.2)	0.37	89.6 (26.2–195.4)	82.7 (44.1–115.2)	0.41
AKI as defined by RIFLE at the first day of admission, No AKI|R/I/Fcc) (%)	0|41/53/6	100|0/0/0	<0.0001	0|23/45/32	100|0/0/0	<0.0001
CKD on admission, CKD‐EPI < 60 mL/min/1.73m^2^ (%)	5.9	11.8	1.00	9.1	11.8	1.00
Mortality rate, after 3 mo/1 y/2 y (%)	35.3/35.3/35.3	29.4/52.9/64.7	0.04	31.8/45.5/59.1	23.5/41.2/52.9	0.84
Need for RRT during ICU (%)	11.8	0.0	0.48	15.9	0.0	0.17
Sepsis stage, sepsis/severe sepsis/septic shock (%)	0/18/82	0/59/41	<0.0001	11/41/48	0/53/47	0.002

CKD, chronic kidney disease; eGFR, estimated glomerular filtration rate; ICU, intensive care unit; MDRD, modification of diet in renal disease; RIFLE, risk/injury/failure/loss of kidney function/end stage renal disease; RRT, renal replacement therapy.

a) Given as mean (range).

b) Two‐tailed probability for continuous data and significance level by Fisher's exact test or chi‐square test for categorical data.

c) **R**isk/**i**njury/**f**ailure of kidney function.

Urine sample pools from ten normal individuals, seven septic patients without AKI and seven septic patients with AKI were repeatedly analyzed at one defined time point. Replicates of these sample pools were included in the training phase to identify analytically robust AKI markers and for variance stabilization of the MALDI marker pattern. During the test phase, replicates of these sample pools were used to determine test precision and to calculate the variance of a negative and positive test result. Another urine pool from seven AKI case and seven non‐AKI control patients was analyzed weekly over an 11‐week period to determine long‐term reproducibility of the MALDI test. Finally, four case and control samples were analyzed daily to evaluate reproducibility of individual samples.

### 2.2 Sample preparation for MALDI‐MS

Urine samples were prepared as previously described 27. Serial dilutions of each sample, which covered two orders of magnitude (0.35–0.0035 μL equivalents of urine) were prepared with 0.1% TFA. Protein concentration was determined by Bradford analysis to be in the range of 13–35 ng/μL. Twelve dilutions were then spotted on a 384 well MTP Anchorchip (Bruker Daltronics, Bremen, Germany) target plate in quadruplicate 28. One microliter of sample was left to dry on the target plate, followed by 1 μL of a 5 mg/mL of α‐cyano‐4‐hydroxycinnamic acid (αCHCA) matrix (Laser Biolabs, Sophia‐Antipolis, France). The matrix concentration was previously determined to be the optimum using standard urine dilution series. No internal standards were added for quantification purposes as this has been shown to be ineffective 28.

### 2.3 MALDI MS analysis

Untargeted MALDI‐MS peptide profiling was performed on a Shimadzu Axima Confidence (Kratos, Manchester, UK) mass spectrometer in reflectron‐positive ion mode. For each sample spot, 36 profiles with 50 laser shots/profile and a laser power between 50 and 56 were carried out. The laser repetition rate was set to 50 Hz that together with an ion gate of 800 Da allowed the detection of peptide signals over a mass range of 100–4000 *m/z*. For peak processing, the peak clean up settings were based on a peak width of five, a Gaussian smoothing filter width of two and a baseline subtraction filter width of six. The peak detection method utilizes a 25%‐centroid arched threshold, an offset of 0.2 mV and a onefold response factor. Monoisotopic peak picking was performed by the Poisson peptide method 29 with a peak picking minimum and maximum mass of 800 and 3900 *m/z*, respectively. External mass calibration using bovine serum albumin peptides (Life Technologies) as standards was performed every four spots. For the eight BSA peptides (mass range: 927.493–2045.028 *m/z*) the maximum allowed mass accuracy variation was adjusted to 10 ppm.

### 2.4 MALDI‐MS data processing, data merging, and regression analysis

MALDI‐MS data were exported as peak lists using Shimadzu's MALDI‐MS Launchpad v2.9.3 software. MALDI‐MS peak intensity data were analyzed in fully automated mode using an improved version (version v5d) of the IAMA software 28. Briefly, all sample data were automatically loaded into spreadsheets and normalized by tallying the intensities by each sample and then dividing each value by the total intensity. Technical replicates were combined using a minimum of three observed peaks per sample and peak as threshold. A regression line was calculated based on the dilutions of every single original sample for every single peak. The algorithm was set to remove any outliers from the dilution rows until a minimum of 20 was reached by recalculating the regression line and the perpendicular distance of each data point from the regression line. Peaks that were not observed in at least three dilution samples per original sample were automatically rejected. Only peak data dilution series resulting in regression lines with a negative slope were passed. The output was set to report the projected values using a dilution factor of one for each peak in every original sample, and the associated statistical output such as coefficient of determination (*R* ^2^), *F* distribution and standard errors were suppressed.

### 2.5 Biomarker definition and support vector machine (SVM) model generation

*p* values for peptide distribution differences between the AKI case and non‐AKI control training groups of sepsis patients were calculated based on natural logarithm transformed intensities and the Wilcoxon rank sum test. A SVM‐based classification model was constructed with a preceding feature selection step based on cross‐validation using the MosaCluster software as previously described 30.

## 3 Results

### 3.1 Patient characteristics and clinical data

We analyzed urine samples from 95 out of 195 randomly selected sepsis patients from the clinical trial NCT01981993 by MALDI MS. The demographic and clinical data of the AKI case and non‐AKI control patients included in the training and test sets is presented in Table 1. Only the severity of sepsis showed markedly significant differences between the AKI and non‐AKI groups in both the training and test sets. For the 95 randomly selected patients, the same characteristics as for the whole 195 patient set of the clinical trial were observed for the frequency of AKI occurrence (*p* = 0.60), gender (*p* = 0.37), age (*p* = 0.47), and serum creatinine levels at baseline (*p* = 0.32).

### 3.2 MALDI analyses and MALDI‐MS data processing

Each urine spot on the MALDI plate produced on average 5000 ± 105 features that were filtered to gain a total list of 1149 peptides in the mass range of 800–3100 Da with a frequency distribution in MALDI samples of >10%. In the subsequent statistical analysis for biomarker selection the number of MALDI‐MS‐identified peptides was further restricted to a number of 937 with a frequency distribution >25% in at least one of the AKI and non‐AKI patient groups.

### 3.3 Peptide marker identification and multimarker model generation

Out of the 51 peptides that were significant in Wilcoxon rank sum statistics 39 were selected via cross‐validation. Preferentially those markers were selected that could be detected with low amplitude variability in the 12 replicates of the sample pools. The biomarker model developed by this strategy is presented in Table 2. Based on these 39 peptides, a SVM classifier was constructed in the 17 AKI case and non‐AKI control samples of the training set including also 12 replicates of the urine sample pools.

**Table 2:** MALDI‐MS analytical and statistical distribution characteristics of the 39 peptides included in the urine peptide pattern for early detection of AKI in the training cohort of sepsis patients

MALDI peptide Ida)	Experimental MALDI‐MS mass mean ± SD [Da]	MALDI statistics		Peptide distribution in AKI case samples of the training cohort (*n* = 21)		Peptide distribution in non‐AKI control samples of the training cohort (*n* = 25)		Direction of regulation AKI vs. non‐AKI		CE‐MS statistics	
		*p* b)	AUC	Mean amp. ± SDc)	Distribution frequency [%]	Mean amp. ± SDc)	Distribution frequency [%]	Fold change of mean amplitudes	Fold change of distribution frequencies	*p* b)	AUC
76	811.097 ± 0.008	3.87 × 10^–2^	0.60	570 ± 833	39	909 ± 1012	60	0.63	0.65		
105	816.548 ± 0.008	6.87 × 10^–3^	0.64	711 ± 931	47	1121 ± 1037	75	0.63	0.63		
214	844.507 ± 0.038	2.35 × 10^–3^	0.65	698 ± 878	47	1150 ± 920	75	0.61	0.63	4.93 × 10^–2^	0.56
273	859.483 ± 0.015	2.88 × 10^–2^	0.61	580 ± 746	42	951 ± 978	63	0.61	0.67	3.31 × 10^–2^	0.56
278	861.026 ± 0.021	1.39 × 10^–2^	0.62	650 ± 1071	38	1012 ± 1263	65	0.64	0.58		
345	878.762 ± 0.021	1.07 × 10^–2^	0.63	830 ± 1022	51	1236 ± 956	77	0.67	0.66		
368	883.695 ± 0.054	1.99 × 10^–2^	0.62	1072 ± 1125	62	1653 ± 1481	81	0.65	0.77		
474	917.227 ± 0.023	3.20 × 10^–2^	0.61	755 ± 783	55	1069 ± 779	75	0.71	0.73		
557	950.624 ± 0.013	1.04 × 10^–2^	0.63	803 ± 812	53	1214 ± 776	77	0.66	0.69		
601	969.401 ± 0.006	3.87 × 10^–3^	0.65	598 ± 681	46	1001 ± 685	73	0.60	0.63		
617	975.372 ± 0.016	3.81 × 10^–2^	0.61	808 ± 792	55	1093 ± 737	75	0.74	0.73		
687	1013.438 ± 0.010	3.22 × 10^–2^	0.61	978 ± 827	62	1196 ± 619	83	0.82	0.75		
715	1025.931 ± 0.030	2.33 × 10^–4^	0.69	715 ± 835	47	1357 ± 902	81	0.53	0.58		
749	1041.460 ± 0.007	2.33 × 10^–2^	0.61	672 ± 720	50	940 ± 665	69	0.71	0.72		
838	1082.065 ± 0.064	2.79 × 10^–3^	0.65	747 ± 816	50	1246 ± 797	81	0.60	0.62		
862	1094.760 ± 0.008	1.38 × 10^–2^	0.62	483 ± 556	46	324 ± 517	31	1.49	1.48		
937	1131.830 ± 0.015	4.97 × 10^–2^	0.60	925 ± 679	70	1124 ± 658	79	0.82	0.89		
1016	1177.712 ± 0.05	1.63 × 10^–3^	0.66	535 ± 642	45	878 ± 651	69	0.61	0.65		
1020	1180.520 ± 0.006	2.48 × 10^–3^	0.64	688 ± 715	51	334 ± 655	23	2.06	2.22	3.15 × 10^–2^	0.57
1077	1212.565 ± 0.007	4.70 × 10^–2^	0.60	497 ± 654	39	722 ± 655	58	0.69	0.67		
1095	1221.740 ± 0.053	1.92 × 10^–2^	0.61	482 ± 678	36	769 ± 655	62	0.63	0.58	2.64 × 10^–2^	0.54
1116	1236.580 ± 0.024	4.23 × 10^–2^	0.60	405 ± 631	31	664 ± 793	46	0.61	0.67		
1148	1255.570 ± 0.006	1.76 × 10^–2^	0.60	577 ± 622	49	783 ± 624	67	0.74	0.73		
1149	1255.774 ± 0.013	4.47 × 10^–2^	0.60	872 ± 696	64	619 ± 738	44	1.41	1.45		
1165	1265.574 ± 0.006	3.30 × 10^–2^	0.61	641 ± 645	51	813 ± 638	65	0.79	0.78	2.14 × 10^–2^	0.61
1226	1307.620 ± 0.013	4.85 × 10^–2^	0.60	640 ± 849	41	945 ± 872	63	0.68	0.65		
1232	1310.609 ± 0.008	6.41 × 10^–3^	0.64	909 ± 691	66	589 ± 695	44	1.54	1.50		
1237	1315.670 ± 0.049	2.60 × 10^–2^	0.60	396 ± 608	31	679 ± 690	52	0.58	0.60		
1245	1323.625 ± 0.028	7.46 × 10^–3^	0.63	661 ± 646	54	357 ± 559	31	1.85	1.74		
1310	1368.637 ± 0.006	1.17 × 10^–3^	0.64	639 ± 769	43	224 ± 509	17	2.85	2.53		
1346	1397.930 ± 0.058	6.23 × 10^–3^	0.62	525 ± 634	43	643 ± 629	54	0.82	0.80	3.96 × 10^–2^	0.54
1374	1423.990 ± 0.085	4.92 × 10^–2^	0.59	372 ± 592	30	614 ± 687	48	0.61	0.63		
1376	1424.670 ± 0.008	1.49 × 10^–3^	0.64	619 ± 752	43	215 ± 497	17	2.88	2.53		
1396	1439.606 ± 0.060	1.28 × 10^–2^	0.62	755 ± 675	58	439 ± 628	35	1.72	1.66	3.79 × 10^–2^	0.59
1543	1576.740 ± 0.007	1.48 × 10^–2^	0.60	359 ± 566	31	195 ± 426	19	1.84	1.63		
1544	1577.740 ± 0.007	3.83 × 10^–2^	0.59	397 ± 556	36	217 ± 474	19	1.83	1.89		
1625	1669.820 ± 0.028	9.00 × 10^–3^	0.61	452 ± 638	39	191 ± 455	17	2.37	2.29	1.01 × 10^–2^	0.59
1636	1676.785 ± 0.007	6.25 × 10^–4^	0.64	632 ± 825	41	172 ± 467	13	3.67	3.15		
1835	2048.950 ± 0.005	1.19 × 10^–2^	0.60	308 ± 542	28	44 ± 185	6	7.00	4.67	4.08 × 10^–2^	0.57

AUC, area under the curve; ID, identifier; MALDI, matrix‐assisted laser desorption ionization; MS, mass spectrometry; SD, standard deviation.

a) Peptide identification number.

b) Wilcoxon *p*‐value.

c) Including zero values.

For the nine peptides identified by cross‐reference in CE‐MS peptide profiles the p value of the Wilcoxon rank sum test and AUC for the AKI versus non‐AKI group difference on the same training cohort of sepsis patients is also given.

### 3.4 ROC analysis and model validation

**Figure 1:**
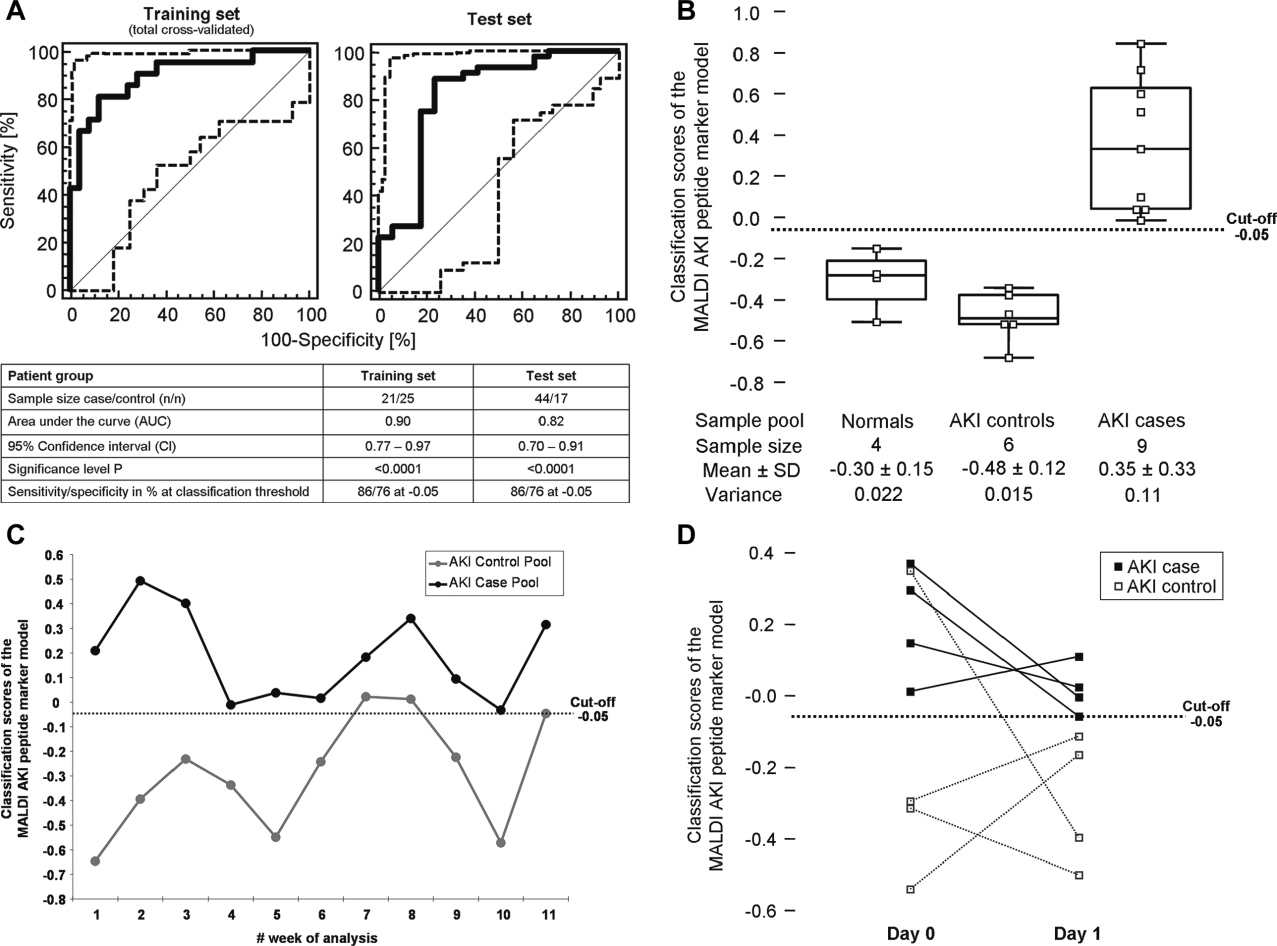
Classification performance characteristics of the MALDI marker pattern for detection of AKI in sepsis patients. (A) ROC curve for the training set of 17 AKI case and 17 non‐AKI control samples after total cross‐validation (left panel). The 12 replicates of urine sample pools from normal controls, AKI cases and AKI controls (four of each pool) that were used in the training phase for variance stabilization of the classifier were also included in this ROC analysis. ROC curve for the independent test set consisting of 44 AKI case (with balanced distribution of sepsis and AKI stages) and 17 control samples (right panel). The point estimates of the model's classification scores at different thresholds are given as thick continuous lines, whereas the 95% confidence intervals (CI's) are indicated by thin dashed lines. The table presents the quality characteristics of the ROC analysis for both the training and test sets. For *P*‐value calculation the departure of the classifiers AUC from 0.5 (a random classifier) was assessed by using a standard *t*‐test. (B) Box‐and‐Whisker representation of the classification scores for the 19 replicates of urine sample pools of normal controls, AKI cases and AKI controls used in the studies validation phase to evaluate the models classification stability and to calculate the variance in the probability distribution of a negative and positive test result for these patient groups by the MALDI marker pattern. (C) Time‐resolved diagram for repeated classification of an AKI control and AKI case sample pool in weekly intervals over an 11‐week period. (D) Dot line diagram of four individual AKI cases and AKI controls analyzed one day apart to evaluate the reproducibility of the sample preparation and instrumentation. Duplicates of the individual samples are connected by a dashed line. In (B) to (D) the classification cut‐off at –0.05 is shown as dashed line.

The model's accuracy to distinguish between AKI cases and non‐AKI controls was first evaluated by receiver operating characteristics (ROC) analysis after leave‐one‐out total cross‐validation on the original training set consisting of 17 AKI case and 17 non‐AKI control patients and by including 12 replicates of the sample pools. This resulted in an area under the ROC curve (AUC) value of 0.90 (95% confidence interval (CI): 0.77–0.97; *p* < 0.0001). At a classification threshold > –0.05, 14 out of 17 AKI case samples and all four replicates of the AKI patient pool scored positive, whereas 13 out of 17 non‐AKI controls and six from eight replicates of the non‐AKI patient and normal control pools scored negative for AKI. This resulted in sensitivity and specificity values of 86% and 76%, respectively (Fig. 1A). The criterion for selection of –0.05 as cut‐off was based on the rationale to obtain the highest level of sensitivity (>85%) at a still acceptable level of specificity (>75%). The model was subsequently tested in the independent validation set consisting of 17 non‐AKI controls and 44 AKI cases. In the validation set the AUC was 0.82 (95% CI: 0.70–0.91; *p* < 0.0001) that confirms that the peptide marker model is highly significant for AKI leading to a sensitivity of 86% and a specificity of 76% at the predetermined cut‐off of –0.05 (Fig. 1A). In comparison, the AUC's for the classical parameters estimated glomerular filtration rate and serum creatinine levels at baseline in this test set of patients were 0.58 and 0.59, whereas absolute urinary NGAL levels and urinary NGAL‐to‐creatinine ratios resulted in AUC's of 0.74 and 0.68, respectively.

In order to test the models classification stability the variance of a negative and positive test result was determined using in total 19 replicates of the urine sample pools of normal controls and of patients with or without AKI (repeatability analysis). As presented in Fig. 1B, the variance for all sample pools is in an acceptable range leading to no misclassification. Along the same line, we were able to correctly classify all replicates of the AKI case pool in the 11‐week lasting weekly analysis series. There were two misclassified control replicates; the false‐positive rate over the 11‐week period was therefore 18% (Fig. 1C). The dot and line diagram presented in Fig. 1D shows the classification scores of duplicate analysis of eight individual samples, four from AKI case and four from non‐AKI control patients at two consecutive days. In this analysis, there was one false positive and no false negative classification.

**Figure 2:**
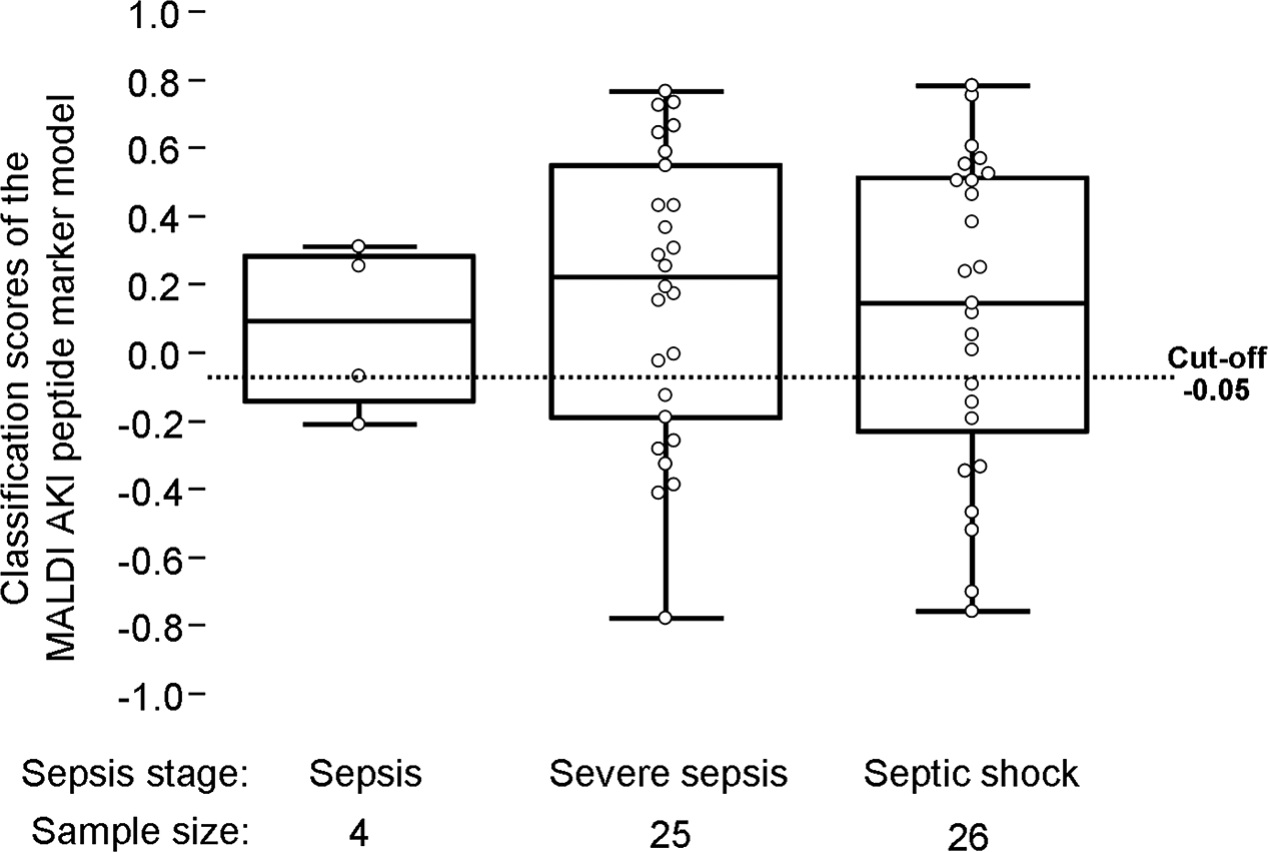
Dependency evaluation of AKI classification by the MALDI marker pattern from the severity grade of sepsis on the test set patient cohort (*n* = 61). The distribution of the AKI models classification scores within the groups of sepsis, severe sepsis and septic shock are given as Box‐and‐Whisker plots. Sepsis staging was performed according to the American College of Chest Physicians/Society of Critical Care Medicine Consensus Conference guidelines. Six patients were excluded due to missing sepsis severity grades. Differences between the groups were found to be not significant in a Kruskal–Wallis rank sum test.

**Figure 3:**
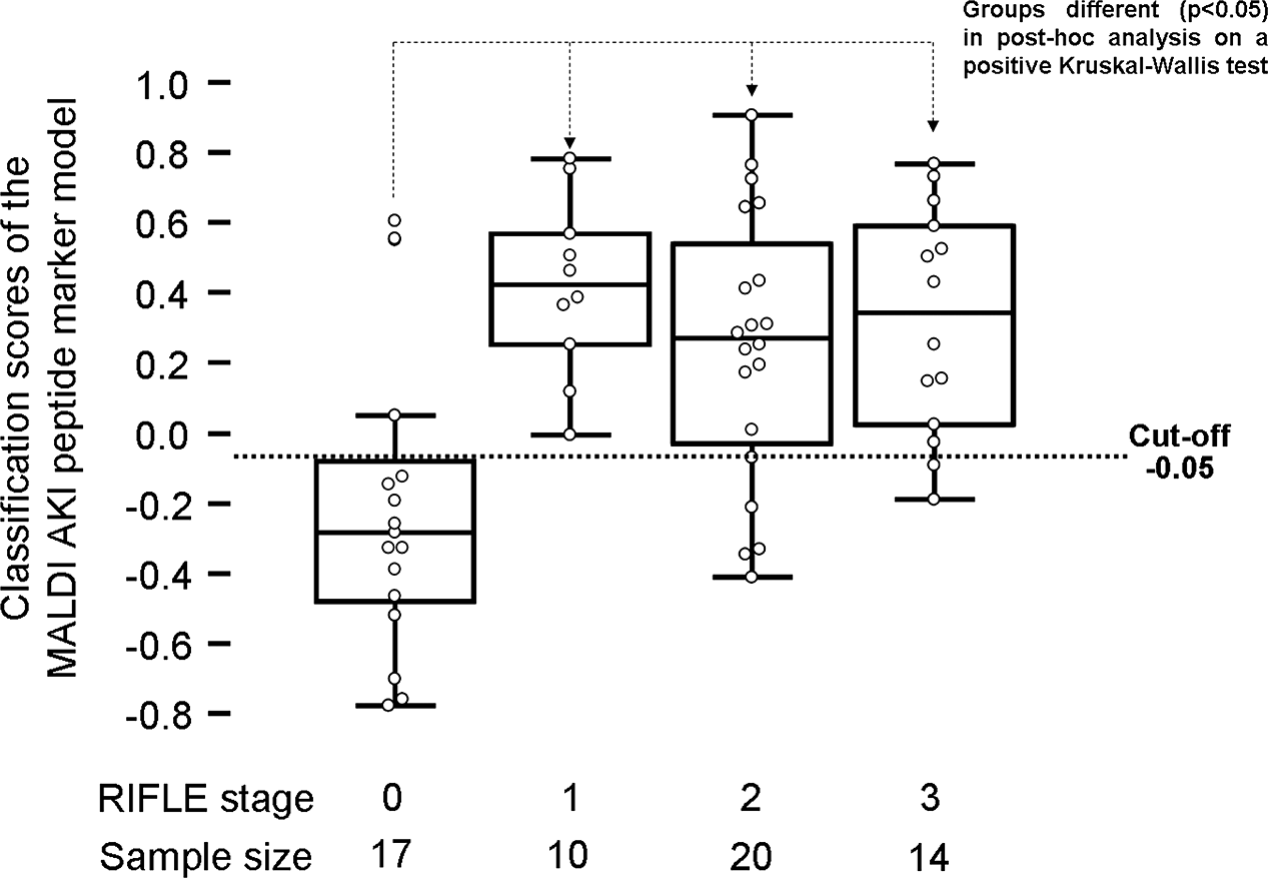
Dependency evaluation of AKI classification by the MALDI marker pattern from the severity of AKI on the test set patient cohort (*n* = 61). The distribution of the AKI models classification scores within the AKI groups of risk (stage 1), injury (stage 2), and failure (stage 3) according to the RILFE criteria together with the non‐AKI control group (stage 0) are given as Box‐and‐Whisker plots. Differences between the groups were found significant between stage 0 and the stages 1–3 in a Kruskal–Wallis rank sum test.

The sepsis stage is not a confounding factor for AKI detection by MALDI (Fig. 2, Kruskal–Wallis rank sum test). As presented in Fig. 3, the MALDI marker model correctly predicted the onset of AKI without rank differences between the RIFLE stages 1–3.

### 3.5 Sample size justification

Assuming normal distribution of the AUC value it was estimated that inclusion of 17 AKI case and non‐AKI control patients in the training set was sufficient to show superiority of the proteome marker model (AUC of 0.82) in comparison to the classical parameters estimated glomerular filtration rate (AUC of 0.58) and serum creatinine levels at baseline (AUC of 0.59) in the test set at a 5%‐level of type one errors and 80% statistical power.

### 3.6 Peptide sequence identification

Direct sequencing of peptides in the MALDI MS relies on mass accuracy of the data alone. However, by also analyzing the samples using CE‐MS, additional information, which adds to the confidence in matching peptides to previously identified sequences can be obtained.

Therefore, the samples were also analyzed by CE‐MS and the CE‐MS peptide profiles specifically examined for the occurrence and distribution of the 39 MALDI marker peptides. From the peptides included in the MALDI marker pattern, nine could also be identified to be differentially regulated in CE‐MS peptide profiles with *p*‐values below 0.05 in the respective Wilcoxon rank sum‐based statistical group comparison. Besides validation of the peptide markers on another MS platform, matching of the peptides to the 2D CE‐MS profiles carries the additional advantage that with the CE migration time an additional peptide‐characteristic could be determined for the nine AKI marker peptides detected by both MS methods. This is especially advantageous for the identification of the amino acid sequence of the peptides since the CE migration time is related to the number of positive charges and allows by the specific line pattern of a CE‐MS spectrum the calculation of the number of basic amino acids inside the peptides amino acid sequence. Via CE‐MS cross‐reference and by a search in our peptide sequence database 27, it was possible to resolve the sequence of seven out of the 39 peptides included in the MALDI marker pattern. The peptides from the MALDI AKI marker pattern of which the amino acid sequences could be retrieved by this strategy are presented in Table 3.

**Table 3:** List of sequence‐identified peptide markers in the MALDI‐MS AKI detection model

MALDI peptide Ida)	MALDI‐to‐CE‐MS peptide matching		CE‐MS statistics		MS/MS amino acid sequence information				
	Experimental MALDI‐MS mass mean ± SD [Da]	Experimental CE‐MS mass mean ± SD [Da]	*p* b)	AUC	Sequencec)	Protein name	AAd)	Sequence‐derived mass [Da]	Sequence‐specific peptide identifier
**214**	844.507 ± 0.038	844.447 ± 0.019	4.93E‐02	0.56	NGERIEK	β‐2‐microglobulin	62–68	844.440	B2M[Asn^62^‐Lys^68^]
**1020**	1180.520 ± 0.006	1180.548 ± 0.022	3.15E‐02	0.57	GppGppGPAGKEG	Collagen α‐1(I) chain	893–905	1180.536	COL1A1[Gly^893^‐Gly^905^]
**1095**	1221.740 ± 0.053	1221.571 ± 0.025	2.64E‐02	0.54	IGPpGPAGApGDKG	Collagen α‐1(I) chain	769–782	1221.599	COL1A1[Ile^769^‐Gly^782^]
**1165**	1265.574 ± 0.006	1265.587 ± 0.012	2.14E‐02	0.61	SpGPDGKTGPpGPA	Collagen α‐1(I) chain	546–559	1265.589	COL1A1[Ser^546^‐Ala^559^]
**1396**	1439.606 ± 0.060	1439.655 ± 0.015	3.79E‐02	0.59	TIDEKGTEAAGAMF	α‐1‐antitrypsin	363–376	1439.660	SERPINA1[Thr^363^‐Phe^376^]
**1625**	1669.820 ± 0.028	1669.681 ± 0.025	1.01E‐02	0.59	DEAGSEADHEGTHSTK	Fibrinogen α chain	605–620	1669.682	FGA[Asp^605^‐Arg^621^]
**1835**	2048.950 ± 0.005	2048.922 ± 0.024	4.08E‐02	0.57	TGPAGEpGREGSPGADGPPGRD	Collagen α‐1(II) chain	1028–1049	2048.915	COL1A2[Thr^1028^‐Asp^1049^]

AA, amino acid; AUC, area under the curve; CE, capillary electrophoresis; Da, Dalton; ID, identifier; MALDI, matrix‐assisted laser desorption ionization; MS, mass spectrometry; SD, standard deviation.

a) Peptide identification number.

b) Wilcoxon *p*‐value.

c) Lower case *p* indicates hydroxyproline.

d) Amino acid position according to UniProt Knowledge Base numbering.

For sequence assignment, the MALDI‐MS peptide markers were matched to already sequence‐identified peptides of the same mass range that demonstrated statistically significant group differences in CE‐MS analysis of the same training cohort of sepsis patients on whom the AKI MALDI model was originally established.

### 3.7 Comparison to CE‐MS‐based classification

For a subset of 36 AKI case and 16 non‐AKI control samples peptide profiles from both MALDI and CE‐MS are available. Classification of this subset by the 20‐peptide marker panel for AKI prediction in CE‐MS resulted in only poor classification performance with the AUC being at 0.55 (95% CI: 0.40–0.69). In contrast, a better differentiation of AKI versus non‐AKI in this sepsis patient cohort was possible if the CKD‐specific CE‐MS model was applied, the AUC being at 0.79 (95% CI: 0.65‐0.89). In the case were the MALDI and the CKD CE‐MS classifier were combined by logistic regression an AUC of 0.94 (95% CI: 0.83–0.99) was obtained resulting in a sensitivity and specificity of 92 and 88% at the Youden index of 0.54 as logistic regression score.

## 4 Discussion

There is a clinical need for new biomarkers to allow for a more accurate and timely detection of AKI in order to improve short and long‐term outcome. In septic AKI, with no clear timing of renal insult, a panel of biomarkers might be more appropriate to capture this complex pathophysiological process and to allow for a more accurate and early diagnosis compared to serum creatinine and urinary output. We identified a marker panel of 39 peptides that was highly predictive of AKI in sepsis by using a MALDI‐MS platform.

Establishment of the MALDI peptide marker panel was based on a SVM learning algorithm. Briefly, SVMs for supervised learning recognize patterns, which they can transform to numerical membership values. Given a set of training data, marked as belonging to one of two classes (here the AKI case and the AKI control group), the SVM training algorithm builds a high‐dimensional parameter space with one dimension represented by one peptide marker (*n* peptides thus equals *n* dimensions). Within this *n*‐dimensional space, the samples are ordered according to the peptide's log‐transformed amplitudes and a separation hyperplane is drawn by maximizing the margin between opposite positions of the case and control data points. After establishment of the SVM classifier, new samples are assigned to either the case or control group according to the degree of similarity in their peptide marker profiles. SVM‐based multimarker models have shown promising results, and we previously demonstrated that by using a panel of 20 peptides we could improve classification of AKI versus non‐AKI ICU patients with high sensitivity and specificity 19. However, the CE‐MS platform used in this study is currently not suited for daily clinical practice. MALDI‐MS theoretically offers the advantage of high throughput, is fast and cost effective and does not require highly trained staff to operate it.

MALDI‐MS has previously been used to detect biomarkers for diagnosis and prognosis of a range of clinical disorders such as hepatocellular carcinoma, hepatoblastoma, and pancreatic cancer 31, 32, 33. Furthermore, MALDI instrumentation is becoming more widely available in clinical settings for rapid identification of pathogens 21. However, a number of studies have identified a range of problems with data quantification and reproducibility of results in protein studies 34, 35, questioning the advantages of this platform for clinical proteomics use.

We have previously reported on a method to improve the relative quantification of MALD‐MS analysis for proteomic biomarker assessment 28. A further development on the method used in the original MALDI study was the elution of the peptides from the desalting columns in 0.1% TFA, allowing direct spotting onto the MALDI plate for immediate analysis. The technique of spotting a range of dilutions of the sample onto the MALDI plate and then using an algorithm to identify the linear range of individual peptides for quantification provides a simple solution to the aforementioned problems. In addition, the software for quantitative analysis by MALDI was further developed to allow automated data evaluation. In AKI samples from patients with sepsis, we developed a biomarker model made up of a panel of 39 urinary peptides. Applying this model to the test set containing 44 cases and 17 controls produced an AUC of 0.82 (*p* < 0.0001) with a sensitivity of 86% and specificity of 76%. Although CE separation of peptides prior to MS analysis would result in higher numbers of detected peptides and would allow via lower interference of signal peaks for a better relative quantification of single peptides 28, 36, the MALDI‐MS biomarker developed here compares well alongside the state of the art CE‐MS biomarkers.

During development of the MALDI test, it became obvious that replicates of AKI case and non‐AKI control sample pools had to be included to ensure reproducibility in sample classification. This was a crucial step in respect to identify the most robust peptide markers and to variance stabilize the multimarker model in the SVM learning phase.

Also of great interest was to determine how well classification by the MALDI‐MS marker pattern is associated to the assessment of the patients on admission with their RIFLE scoring. With a cut‐off value of –0.05, the MALDI‐MS marker is correctly predicting AKI even when the RIFLE assessment score is at stage 1.

Only seven out of the 39 peptides making up the model had sequence information available. The identified peptides are fragments from the collagen chains alpha‐1(I) (COL1A1) and alpha‐1(II) (COL1A2), alpha‐1‐antitrypsin (SERPINA1), beta‐2‐microglobulin (B2M), and fibrinogen alpha chain (FGA). In comparison to the 20‐peptide marker pattern for AKI prediction in CE‐MS 19, COL1A1, SERPINA1, B2M, and FGA could be reproduced by MALDI as AKI‐specific peptide marker source. Direct comparison of the two marker patterns revealed identity of the SERPINA1‐derived peptide SERPINA1[Thr363‐Phe376], whereas the COL1A1‐, B2M‐, and FGA‐derived AKI peptide markers in MALDI are for the most part smaller fragments of the previously described CE‐MS‐identified peptide markers. This accounts for the peptides B2M[Asn62‐Lys68], COL1A1[Ser546‐Ala559] and FGA[Asp605‐Lys620] in MALDI with B2M[Leu60‐Ser81], COL1A1[Thr541‐Gly560], and FGA[Asp605‐Arg621] as CE‐MS counterparts. In this context, it must be noted that the detected mass range in MALDI‐MS is generally more restricted to smaller sized peptides (0.8–3.2 kDa) compared to the one in CE‐MS (0.8–16.0 kDa).

The seven MALDI AKI peptide markers known by sequence were also compared to the 273 CKD‐peptide markers described by Good et al. 27. In this case, there was an overlap of three peptides affecting the peptides SERPINA1[Thr363‐Phe376], COL1A1[Ser546‐Ala559], and FGA[Asp605‐Lys620].

In accordance to our previous study on AKI peptide markers in urine by CE‐MS 19, further support is given to the hypothesis that increased urinary levels of peptide fragments derived from the blood proteins B2M and SERPINA1, and decreased urinary levels of FGA are early signs of AKI. With regard to the collagen alpha‐1(I) and alpha‐1(II) chain structural proteins, peptide fragments thereof were found to be either down‐ or upregulated in AKI compared to non‐AKI sepsis patients. Differential excretion of collagen alpha chain‐derived peptide fragments was already described in a number of other proteomic studies on CKD and AKI 19, 27, 37, 38 and is indicative for alterations in extracellular matrix turnover 39, 40, i.e. via tumor necrosis factor alpha‐induced activation of matrix‐degrading proteases 41, 42. Alterations in extracellular matrix turnover in turn were interpreted as a possible trigger factor in the development of CKD 43. Therefore, the peptides identified in this study link particularly well to the current pathophysiological concept of AKI that might explain their good performance—as a panel—to predict AKI.

Efforts are now underway to resolve the amino acid sequence of all remaining unidentified peptide markers by postsource decay mass spectrometry. If the multimarker model is only restricted to the sequence identified peptide markers this resulted in only moderate classification performance with the AUC being 0.72 (95% CI: 0.59–0.83) demonstrating the requirement to also include the yet unidentified peptides in the classification model.

The use of MALDI‐TOF analysis for clinical biomarker assessment has not previously been possible due to the poor quantitative nature of the technique 44. We previously presented methodology and software solution to these problems 28 and now present data on their application to biomarker model development. The 39‐peptide marker panel of sepsis‐induced AKI provides a significant improvement in sensitivity and selectivity over single marker tests.

In contrast to the MALDI AKI peptide marker pattern, our previously established 20‐peptide marker pattern for AKI prediction of CE‐MS peptide profiles failed to reliably detect AKI in this cohort of sepsis patients. Since when applying CE‐MS a CKD‐specific peptide marker profile enabled better differentiation of AKI from non‐AKI, we hypothesize that in the study cohort of sepsis patients those with AKI more likely contain features related to the CKD rather than the AKI CE‐MS peptide profile. Even in the initial phase, pathways related to CKD might therefore be active in this sepsis patient collective. According to the finding that the combination of the MALDI and CKD CE‐MS tests by logistic regression significantly improves classification accuracy, a strategy where MALDI‐positive patients are subsequently reassessed by the CKD CE‐MS classifier might be considered in the future to further improve diagnosis of septic AKI and its progression to CKD in the clinical setting.

The limitation of the study is that it describes a proof‐of‐concept study on a relatively small population of 95 sepsis patients evaluating if an AKI‐specific peptide marker pattern with performance characteristics similar to the one already established for CE‐MS is also possible for MALDI‐MS peptide profiles. For this specific aim, the group of selected patients is well defined and the diagnosis of AKI by RIFLE is based on both the serum creatinine and the urinary output criterion. A historical baseline serum creatinine value was available for all patients.

The analytical reproducibility of MALDI analysis in a clinical setting has been questioned and discussed 44. To access the long‐term stability of the biomarker model and also to address the MALDI instrumentation reproducibility we undertook to analyze pools of samples over an 11‐week period. In this series, we correctly classified all of the AKI case pool replicates. Two AKI control replicates in the 11‐week series were classified positive, which resulted in a false‐positive rate of 18%. Although this is still evaluated to be in an acceptable range, efforts were made to identify the reason for this tendency toward false‐positive classification. We found that despite frequent calibration checks, the laser output power in the instrument varied over time and was not consistent at the settings selected within the method file. An increase in laser output can increase the noise level in the signal to a point where "noise" peaks were recorded as real peptide signals leading to a higher false‐positive rate. Inclusion of the laser output power in the calibration method that is currently under nonmodifiable software control is in our opinion an essential step to further improve the diagnostic accuracy of our MALDI test for its use in a daily clinical setting to repeatedly monitor AKI progression in sepsis patients.

## 5 Conclusion

We have developed and tested a new urinary biomarker model for septic AKI that demonstrates sufficient sensitivity, specificity, and classification stability to allow for significant improvement in timely detection of AKI. The MALDI‐MS platform for this test is similar to the one already available in the nonresearch setting 21, and the timeframe and per sample cost are in keeping with those required for daily clinical use. The next crucial step would be to demonstrate a significant benefit of this biomarker panel in a prospective clinical study, which is currently being planned.

*H.M. is founder and coowner of Mosaiques Diagnostics, Germany and J.M., M.D., and M.P. are employed by Mosaiques Diagnostics. All other authors declare that they have no competing interests*.
